# Clinical Experience in Late Antiquity: Alexander of Tralles and the Therapy of Epilepsy

**DOI:** 10.1017/mdh.2014.27

**Published:** 2014-07

**Authors:** Petros Bouras-vallianatos

**Affiliations:** Centre for Hellenic Studies, King’s College London, Strand, London WC2R 2LS, UK

**Keywords:** Alexander of Tralles, Clinical experience, Epilepsy, Pharmacology, Natural remedies, Popular/folk medicine

## Abstract

Alexander of Tralles, writing in the late sixth century, combined his wide-ranging practical knowledge with earlier medical theories. This article shows how clinical experience is used in Alexander’s works by concentrating on his therapeutic advice on epilepsy and, in particular, on pharmacology and the group of so-called natural remedies. I argue that clinical testing is used not only for the introduction of new medicines but also as an instrument for checking the therapeutic effect of popular healing practices. On another level, this article discusses Alexander’s role as the author of a medical compendium; it suggests that by marking the cases of clinical testing with a set of recurrent expressions, Alexander leads his audience to reflect on his medical authority and personal contribution.

## 1. Introduction

Late antique medical authors have not received much attention from medical historians and specialists working on social and cultural history, and reception studies.^1^ Scholars have considered the preservation of classical texts as the principal contribution made by late antique authors; the ‘refrigerators of antiquity’, as they have been called.^2^ It is only to be expected that, as they used the same language as the classical authors, they had privileged access to this tradition. Any in-depth study of late antique medicine should move beyond the, admittedly true, fact that it preserved the classical medical heritage, and look for important observations linked to medical practice.

Recent studies on the works of Oribasios (AD ca. 325–after 395/6), Aetios of Amida (first half of the sixth century AD), Alexander of Tralles (AD ca. 525–ca. 605) and Paul of Aegina (late sixth century–died after AD 642) on the Greek side or of Caelius Aurelianus (fl. around AD 400), Theodore Priscianus (fourth/fifth century AD), Cassius Felix (fifth century AD) and the author (ca. fourth century AD) of the *Medicina Plinii* on the Latin side, have emphasised the crucial role of these authors in the shaping of a medical tradition and transfer of medical knowledge.^3^ Their medical expertise reinforced their literary skills in selecting the most essential parts of earlier texts and re-arranging an otherwise vast and sometimes chaotic material into a more systematic form. In particular, Alexander of Tralles amalgamates his abilities as a compiler with his own extensive clinical experience,^4^ producing works which are marked by a strong authorial presence. Even though he was much influenced by the theories and practices of Galen and followed a Hippocratic understanding of humoural pathology and physiology, he kept on striving to find new ways of treating disease and researching the effectiveness of his therapies.^5^ He conscientiously cited earlier authorities and did not hesitate to disagree even with Galen when common sense demanded.^6^

Alexander practised in the reign of Justinian I (AD 527–65) and, according to the contemporary historian Agathias, writing in AD 557, came from a well-known provincial family from the city of Tralles, in the region of Lydia in Asia Minor.^7^ His father, Stephen,^8^ was a physician and the most prominent of his brothers was Anthemios,^9^ a distinguished mathematician and best known as the architect of the great church of Hagia Sophia in Constantinople. Agathias states that ‘Alexander settled in Rome, having been called there due to his great prestige’.^10^ So, if Agathias is to be trusted, Alexander became a highly successful and respected physician, who enjoyed recognition among his contemporaries. Thus, Alexander should not be seen as a marginal provincial figure, but as a well-connected member of the sixth-century establishment.

Alexander’s main work, the *Therapeutics*, follows the well-established medical tradition of writing *a capite ad calcem* (from head to toe) and has details on diagnosis and treatment of diseases divided into twelve books (see Table 1).^11^ In addition to *Therapeutics*, two other genuine works survive. These are a treatise *On Fevers* dedicated to his friend Kosmas and a letter *On Intestinal Worms*, which was composed for his friend Theodore.^12^ The works circulated widely and were well received as early as the early seventh century, when they are already being cited by Paul of Aegina. A first translation of Alexander’s works into Latin is estimated to date to around AD 700; translations into Arabic and Hebrew followed.^13^ Alexander must also have written other works that have not survived, such as a book on fractures and wounds, to which there is a reference in his *Therapeutics*.^14^ A work *On Eye-diseases*, found in some manuscripts of the *Therapeutics*, is now considered spurious.^15^ The same applies to a text entitled *On Pulses and Urine*.^16^

**Table 1:** Contents of the *Therapeutics* (Detailed presentation of Book I) according to Puschmann’s edition,*op. cit.* (note 7).

Alexander of Tralles’ *Therapeutics*	Contents	Puschmann, *op. cit.* (note 7)
Book I	Chapters 1–9: Diseases of the hair and scalp	I, 465–83
	Chapters 10–12: Headaches	I, 465–507
	*kephalalgia*	I, 465–83
	*kephalaia*	I, 485–99
	*hēmikrania*	I, 499–507
	Chapter 13: On phrenitis	I, 509–27
	Chapter 14: On lethargy	I, 527–35
	Chapter 15: On epilepsy	I, 535–75
	Chapter 16: On paresis^105^	I, 575–91
	Chapter 17: On melancholy	I, 591–617
Book II	Eye diseases	II, 3–69
Book III	Diseases of the ears and the parotid area	II, 71–125
Book IV	Laryngeal and pharyngeal diseases (*synanchē*)	II, 125–45
Book V	Pulmonary diseases	II, 147–227
Book VI	Pleurisy	II, 229–43
Book VII	Gastric diseases	II, 245–319
Book VIII	Cholera and colics	II, 321–77
Book IX	Hepatic diseases	II, 379–439
Book X	Dysentery and dropsy	II, 439–61
Book XI	Genito-urinary diseases	II, 463–501
Book XII	Gout	II, 501–85

The state of research into Alexander’s works remains at quite a basic level and various aspects are as yet unexplored. As John Scarborough aptly stated: ‘it is quite surprising that a full-fledged study of this magnificent physician has not yet been produced by modern medical historians’.^17^ In this paper, I would like to examine how Alexander attempts to communicate his specialised knowledge to his reader by focusing on the physician’s practical experience. My purpose is not to evaluate the efficacy of his therapeutic methods; instead, I aim to explore Alexander’s experiential claims and highlight certain features of his methods and the presentation of his material. My study focuses on the chapter ‘on epilepsy’ in the *Therapeutics* for two main reasons. First, it is one of the most diverse as regards material, containing a large number of so-called ‘natural remedies’,^18^ and one of the longest chapters in the entire work, occupying twenty of the eighty-nine printed pages that make up the first book of the *Therapeutics*.^19^ Furthermore, since epilepsy was one of the most well-researched diseases in antiquity, there is a plethora of classical and late antique material available that will provide useful data for comparison with Alexander’s approach.^20^ My study delves into two main areas of Alexander’s therapeutic advice. First, I examine the field of pharmacology; next comes the group of natural remedies.

## 2. Peira and Self-promotion Strategy in Alexander’s Works

Proems are traditionally ideal places from which to collect information about the author’s aims and motivations. To provide a convenient starting point, I will refer to the short preface that preceded his work *On Fevers*, which circulated in the manuscripts as part of the last book of the *Therapeutics* together with the treatise *On Fevers*. Alexander states:

‘My beloved Kosmas, I readily obeyed your demand to set out for you what I have assembled from my [ἡμῖν, lit. “our”] rich experience [πείρας] of treatments of various diseases (…) although I am now an old man and no longer able to exert myself greatly, I obeyed and wrote this book, after having collected my experiences [πείρας] from my many contacts with human diseases. And I know that this will please many people, who are not envious to look at the good reasoning [τό τε εὐμέθοδον] of my medical theories [τῶν θεωρημάτων] and at the same time the brevity and clarity of the exposition.’^21^

The author is at an advanced age and gives the impression of a practising physician who has decided to share the fruits of his long, erudite career with future generations. In his introductory declaration, he gives an emphatic pointer using the word *peira* to refer to a central aspect of the way in which he composed his text. *Peira* seems to include all of the accumulated knowledge acquired through contact with his patients.^22^ It also appears to be part of his attempt to claim authority, by presenting himself as a medical practitioner, and not merely as a compiler of medical writings in contrast, for example, to Aetios of Amida, who often simply copies first-person verbs and pronouns from the original source.^23^

Thus, Alexander acknowledges the special nature of the statements related to his clinical activity and takes them to be distinct parts of discourse in his works. These passages may be short, eg. just a few words, or sometimes quite long, covering a couple of lines. For example, he may simply refer to the effectiveness of a certain remedy by the use of a short phrase such as ‘this works wonderfully’.^24^ Nonetheless, in the vast majority of cases his use of the first-person singular is clearly connected with a statement emphasising the role of experience. Alexander is usually presented as transmitting an observation or reporting his thoughts and medical actions. For example, he may use a past form of the verb *theaomai* (to behold) or *heuriskō* (to discover) or even *horaō* (to see/to know).^25^ The narration usually shifts from the first-person indicative to the second- or third-person imperative advising the reader accordingly, eg. ‘give [δίδου] six *grammata* ^26^ (of the aforementioned medicament)’.^27^

The first-person singular was used in the classical period by philosophical, scientific and medical authors in their attempts to engage a reader’s attention when referring to some innovative approach.^28^ In Alexander’s case, it seems to reveal the author’s attempts to promote the quality of his recommendations by providing his own perspective. It is a way of giving his words added force as the direct outcome of an experience, revealing the author’s expectation that his audience will take note of and remember his contribution, when faced with similar situation themselves. In addition to the first-person singular, as we have already seen in relation to Alexander’s proem, he chooses to use the first-person plural, ‘we’, in setting out his experiences.^29^ The use of the first-person plural is common in ancient Greek and Latin scientific texts and Galen himself makes use of it in referring to his clinical activity and in particular in his case histories.^30^ It seems that, in Alexander’s case, the ‘we’ refers to the author and his reader, in turn implying a notion of ‘communality’.^31^ There may be no direct response from the reader, but as has already been noted, Alexander is addressing his friends and seems aware of a contemporary circle of individuals, who had an intense interest in medical matters. Hence, there is an audience, which he attempts to involve in his own world of medical knowledge, which is based on his own records and observations.

## 3. Epilepsy

Having provided an overview of Alexander’s ways in referring to his practical experience, I now turn to discuss his experiential statements in more detail by focusing on a particular chapter of his *Therapeutics*, that is on epilepsy (see Table 2). According to Puschmann’s edition, the chapter ‘on epilepsy’ is divided into thirty-one paragraphs.^32^ Each one has a separate title, which is related to the contents of the paragraph and may be quite long, such as ‘treatment of patients suffering from epilepsy originating from the limbs’ or just a single word such as ‘another (remedy)’.^33^ We can identify three distinct thematic sections. The first and shortest section deals with the pathology of the disease, the next with therapeutics and the last with natural remedies.^34^

**Table 2:** Contents of Chapter 15 of Book I of the *Therapeutics*.

Alexander of Tralles, *Therapeutics*, Book I, Chapter 15	Puschmann, *op. cit.* (note 7)
*First section:*	I, 535–39
Introduction	I, 535–37
Symptomatology	I, 537–39
*Second section:*	I, 539–57
Diagnosis of milk	I, 539–45
Simple drugs for epilepsy	I, 545–47
Purgatives for epilepsy	I, 547–49
Therapeutic advice for epilepsy originating in the stomach	I, 549–51
Therapeutic advice for epilepsy originating in the limbs	I, 551–57
*Third section:*	I, 557–75
Natural remedies	

In the initial section of the chapter, Alexander, following Galen, distinguishes three forms of epilepsy: (a) epilepsy originating in the brain; (b) epilepsy originating in the stomach; and (c) epilepsy originating in the limbs.^35^ He characterises epilepsy as a ‘moist’ and ‘cold’ disease and in his remedies attempts to avoid any concentration of ‘wetness’ in the cerebral ventricles, following the well-established principles of humoural pathology.^36^ A ‘thick’ humour is considered responsible for the accumulation of ‘wetness’, and as in the Galenic approach, this humour may consist of phlegm or black bile.^37^ Furthermore, in the case of those suffering from epilepsy originating in the stomach, it is probable that an additional high concentration of yellow bile could reach the brain and exacerbates the disease.^38^

Alexander is clearly not interested in presenting a detailed symptomatology or arguing about the various stages and types of diseases in his works. His main focus is on therapeutics. When theory is introduced it is only to provide the essential background, which one might require when dealing with the disease. All of the passages which are connected with Alexander’s references to his practice are found in the last two sections of his chapter. Thus, it is certainly not random that there is only one example of the use of the first-person in the first section of the chapter, while in the next two sections we find twelve and eleven examples, respectively.^39^

## 4. Pharmacology

Alexander’s therapeutic approach pays particular attention to pharmacology, which for him constitutes the most dynamic branch of therapeutics.^40^ For example, in his *Therapeutics*, he may sometimes provide dietetic advice and rarely mentions natural remedies, but he makes clear that he is not enthusiastic for the use of surgery: ‘arteriotomy, trephinisation, cauterisation, and all the other remedies (…) become a punishment to many rather than a cure’.^41^ His therapeutic advice on epilepsy starts by concentrating on epilepsy in infancy and the special dietary requirements a wet nurse should follow.^42^ The concept of good quality milk as fundamental measure of successful treatment in cases of infantile epilepsy was not a new one.^43^ Furthermore, the consequences of various foodstuffs as regards the accumulation of a ‘thick’ humour had already been elaborated in detail by Galen.^44^ Alexander does not seem particularly interested in involving any cases from his clinical experience and proceeds to pharmacology.

We know that the field had already provided a great number of available agents for epilepsy. For example, Temkin lists forty-five pharmacological substances mentioned by Dioscorides (fl. middle of the first century AD) in relation to the disease.^45^ Alexander shows that he too has something to add. The part following the dietary advice concentrates on the use of simple drugs for epileptic children. Thus, his account reads as follows:

‘(…) during the winter, the decoction of hyssop^46^ can be very useful. And so many (patients) having used only this (decoction of hyssop) were cured, and they no longer fell ill for a second or third time with the same disease.’^47^

Hyssop had been suggested for the therapy of epilepsy by Greek authors such as Aretaeus (ca. first century AD) and, one of the chief representatives of the Methodic school, Soranus (second half of the first century–early second century AD) in the past.^48^ In his turn, Alexander believes that hyssop is a very efficient drug, which can cure the disease for a long period of time, suggesting an acute therapy with a long-term outcome, which confirms his focus on youth. This is not a simple reference to a pharmacological use, but it probably implies his persistent attempts to check past remedies with real patients. However, it is not representative of Alexander’s particular authorial manner of expressing the outcome of his clinical activity.

The first remarkable instance is found in the next part dealing with purgatives. Alexander starts his account by focusing again on those suffering from the disease from an early age. Purgation, which led to the subsequent evacuation of harmful humours from the patient’s body was an extremely common therapeutic procedure for the majority of human diseases in antiquity.^49^ Alexander refers to various kinds of purgatives, which were used a great deal to draw off noxious humours. His first mention comes in a statement on the effectiveness of an antidote. The use of the first-person singular for the first time in this chapter alerts the reader: ‘I know [οἶδα] many (patients) who were cured by the aforementioned purgative (a composite called *theodōrētos*)’.^50^ By suggesting the use of *theodōrētos*, he is referring to an already known medicament, which he had found to work well when applied to his patients. Here the contact with the disease is reported as a means of testing, and thus adopting an existing pharmacological therapy as with hyssop. However, in the next couple of phrases, we can see Alexander quite openly illustrating his active involvement in making treatment decisions:

‘If, however, the harmful humoural mixture continues to cause problems and the disease persists, then let the pills prepared by myself, from which I have not found [εὗρον] anything stronger, be swallowed. The preparation of the pills is as follows:’


 ounce^51^ aloe^52^


 ounce scammony^53^


 ounce gum^54^


 ounce of colocynth^55^


 ounce bdellium^56^^,^ 57.

Alexander himself appears to prepare a pharmaceutical dosage form, in this case a number of pills, which constitutes the ultimate measure against the on-going humoural imbalance. He goes a step further by suggesting a recipe and providing the quantities of the ingredients in detail. According to his text, he has tested the aforementioned composite drug by comparing its effectiveness with other drugs. His assessment did not depend on certain criteria, *diorismoi*, as laid down by members of the Empirical sect or later even Galen himself in his concept of ‘qualified experience’.^58^ The approach he follows is as eclectic as it needs to be to help his patients effectively.

At a first stage experience is used as a testing device, as in the case of the *theodōrētos* remedy. The next stage is a research process. Alexander’s use of the verb *heuriskō* implies an on-going testing, in which the physician has to try to discover a new recipe by changing the quantities of certain ingredients or adding new ones. This is even more obvious in the next example where Alexander suggests using purgatives on patients who suffer from epilepsy originating in the limbs. Here Alexander chooses to give his advice on a certain purgative by introducing a real scene from his professional career:

‘I beheld [ἐθεασάμην] one of those readers falling down; then, he said, whenever it was about to happen, he felt an aura of cold rising from the tarsus up to the brain. And so I purged him by giving him pills with which to remove the phlegm and the black humour (…) and I [ἡμῶν, lit. ‘we’] have practised in this way, the young man became healthy. The medicament, which I applied in his case to help this treatment was the herb called pepperwort^59^; for although there are other herbs producing the same effect, none is as well suited as that herb.’^60^

Galen reports a similar case in his *On Affected Parts*.^61^ In both cases the patient is young and suffers from epilepsy originating from the lower limbs. In the Galenic example the focus is clearly on symptomatology and there is very little on therapy; Galen succinctly suggests that the patient’s body should be purged with the use of deadly carrot^62^ or mustard^63^ without providing any further details. Although it seems that Alexander follows Galen’s case history at the beginning,^64^ he clearly shows a greater emphasis on therapy, which differentiates his approach from that of his predecessor.

Alexander’s authorial voice is certainly stronger here than in any other part of his text so far. He is the only physician present in the particular event, who manages effectively to save his patient. The reader is transferred to the locus of medical activity and is able to get a closer look at Alexander’s methods. Experience in the form of the first-person singular is used as a device of communication in order to highlight the author’s ability to deal with a demanding situation by suggesting new drugs. But, let us concentrate on the suggested remedy. Its therapeutic approach is expressed through an instant response with the use of pepperwort alone. Although previous medical writings about the therapy of epileptics had not made use of this ingredient, it is reported as a drug with an intensely ‘warm’ quality,^65^ which may alleviate the ‘moist’ and ‘cold’ quality in a patient’s brain. This is indicative of Alexander’s heuristic approach in dealing with disease, which is a process of both testing and discovery.^66^ Alexander appears aware of various purgatives, which may produce a similar effect and thus could have been used to test the efficacy of something previously untried. He finally comes to a logical outcome through a repeatable process, which highlights the special use of a certain pharmacological agent, in this case, pepperwort. However, there is not enough evidence in Alexander’s text as to *why* or *how* one drug is more effective than another.

Later on, he gives an account of a proposed purgation, in this instance for those suffering from chronic epileptic seizures. He starts once again by giving a detailed recipe, here of a purgative based on white hellebore^67^ and followed by certain steps on the process of purgation. Alexander clearly alludes to the ‘cyclical’ therapy of Methodism.^68^ The first phase, the so-called recuperative cycle, aimed to rebuild the patient’s condition through dietetics. The next one, the re-corporative cycle, was dominated by the application of either drastic local drugs or surgery. In the case of epilepsy, Caelius Aurelianus refers to the administration of hellebore during the latter cycle.^69^ However, Alexander has a personal view, providing us with the following sequence of observations:

‘I purged well by using the *hiera* ^70^ and I also gave three *grammata* of Armenian stone^71^ and I helped (the patients). However, I have not found [εὗρον] any other (treatment) so effective in patients with chronic epilepsy, as this purgative (white hellebore) and I know [οἶδα] many who, having been given up by other doctors, were cured by using only this purgative (white hellebore).’^72^

Alexander introduces here a new recipe, which has been compared to two other similar purgatives many times before. He names the drugs, in this case the *hiera* and the Armenian stone, and provides a tangible description of the main components of his ongoing testing process. His own recipe with the white hellebore, however, is presented as the most effective drug for this disease above numerous other purgatives provided by other physicians. The comparison with the latter’s approach highlights competition among contemporary practitioners and is connected with Alexander’s attempts to establish contact with and persuade his reader of the efficacy of his advice. Although his reader cannot easily judge the truth of what he says, this is an element which certainly makes Alexander’s advice seem more authoritative.

Alexander’s eclecticism allows a variety of remedies from a great range of earlier authors to be tried and tested. His procedure follows a similar pattern in all cases; having tested existing remedies devised by various earlier authorities, he attempts to provide a more efficient cure by trying out a closely related or similar drug. To some extent, Alexander’s approach might recall one of the three main epistemological tenets of ancient Empiricism, that is, ‘the transition from like to like’.^73^ Although Alexander seems to have borrowed elements from Empiricists, he does not appropriate their main notions in his approach. The hypothesis deriving from Alexander’s sayings could be expressed as follows: ‘If A does not work on patient X, let us try a similar drug (B). Does B work on patient X?’. The answer can only be ‘yes’ or ‘no’. In the case of composite drugs, the hypothesis could take the following form: ‘If C does not work on patient Y, let us try a variety of C by introducing an ingredient with similar effect or by changing the amounts of the ingredients’. He may sometimes introduce either a new simple or composite drug or devise a new variety of an already known composite drug. This does not, however, amount to the formulation of a wholly new theory on drug activity substantiated by causal explanation. Alexander certainly comes up with significant contributions in purging his patients effectively, but this is not based on scientific experimentation. It contains a simple practical notion, which is enough to show Alexander’s audience his original way of dealing with a disease that has already been much discussed in pharmacological terms.

## 5. Natural remedies (*Physika*)

Alexander’s individual attitude is even more obvious when he decides to include a number of natural remedies, *physika*, in his discussion of epilepsy.^74^ These may refer either to diagnostics or therapeutics and are characterised by their impact in folk medicine. Although Greek authors such as Galen or Soranus and Latin authors such as Celsus (first century AD) or Scribonius Largus (ca. first half of the first century AD) might include references to popular healing practices, they clearly stated that these therapies fell outside professional medicine and rejected their use on most occasions.^75^ On the other hand, it seems that other contemporary medical authors such as Archigenes,^76^ as confirmed by Alexander’s references, or later ones such as Aetios of Amida^77^ and Marcellus^78^ (ca. late fourth century/early fifth century AD) included a considerable number of these remedies in their therapeutics.^79^ However, this is not the place to speculate on Alexander’s testimony as regards Archigenes’ lost work and the validity of his references; rather it is a chance to explore the way Alexander exploited his own experience in using them.

Alexander makes a clear distinction with regard to his natural remedies, using special subheadings. Sometimes, the sections referring to them are quite long as for example in the chapter under examination, but most of the time they constitute only a minor part of his therapeutic approach.^80^ Among the different types of ingredients, which he uses for his natural remedies, we can identify four main categories: plants, animals, minerals and human humours, such as human blood. In the case of epilepsy, his text contains about twenty examples of such remedies. In the cases where Alexander provides a named sources, apart from Archigenes, he refers to a great number of earlier authors as the sources of some of his remedies, including well-known authors such as Asklepiades Pharmakion (ca. second half of the first century AD),^81^ Servilius Damokrates (first century AD),^82^ Xenokrates of Aphrodisias (second half of the first century AD),^83^ Theodore Priscianus,^84^ and little-known authors such as Straton of Beirut (ca. first century AD),^85^ Moschion (ca. first/second century AD),^86^ or even hitherto unknown such as Marsinos of Thrace,^87^ and obscure authors such as Osthanes (ca. first century BC),^88^ and Zalachthes.^89^ Furthermore, he indicates various places across the Mediterranean, where he had discovered remedies, such as Tuscia (a historical region of Italy consisting of modern Tuscany, a large part of Umbria and the Northern Lazio),^90^ Corfu,^91^ Gaul^92^ and Spain.^93^ Therefore, we can see that he drew his information from a wide range of sources, which confirms once more Alexander’s eagerness to evaluate and adopt every possible remedy.

The relevant section in the chapter ‘on epilepsy’ starts with Alexander’s attempt to explain why he mentions such a large number of these remedies:

‘And so these things are said about epileptics, (it is) all we [ἡμεῖς] know and the long experience [πεῖρα] has taught (us). However, since some people rejoice in natural remedies and amulets and they seek to use them, and according to what they say, they succeed in their aim, I thought that it was fitting to give an account regarding these topics for those who are eager to learn, so that the physician will be well provided from all the available sources in order to be able to help patients.’^94^

First he refers to the earlier sections of his chapter mentioning once more the testing of past remedies in order to confirm their efficacy. However, in the next couple of phrases, he seems quite apologetic. His intentions have changed and he makes clear once more that the most important element in his approach is to find effective remedies regardless of their origin. In a similar vein, in his chapter ‘on hiccups’, he is very frank, saying that he will resort to a certain natural remedy only when ‘no therapy of the (medical) art (…) has power’.^95^ Epilepsy was perceived as the demonic disease *par excellence*, probably as a result of the various effects of epileptic seizures.^96^ Therefore, using popular methods of healing to drive a demon out of a person was quite popular at the period.

Alexander does not hesitate to comment on a number of remedies, showing that he has clearly tested them in his professional life. Here he mostly provides advice in the form of short phrases such as ‘this is most marvellous’ as regards a remedy based on a burned ass skull or ‘this is of great value’ referring to an amulet made of jasper, and thus confirming that he had most probably examined them himself.^97^ In arranging his material, however, he seems to give precedence to some of them on the account of their clinical effectiveness. Thus, these favourable comments are expected to influence his readers and lead selecting which natural remedies might be applied to patients.

However, there are two cases, in which Alexander shows a greater engagement in proving their effectiveness. In the first case, he suggests the use of a gladiator’s rag imbued with blood, which has been burnt and mixed with wine. Aretaeus and Celsus refer to the remedy without commenting on it.^98^ Although he also refers to it, Scribonius Largus in his turn accepts such a therapy falls outside professional medicine.^99^ Alexander prefers to use an unknown author named Marsinos of Thrace as his source and supplements the passage with an observation: ‘this has proved many times to be successful through experience [πεῖραν]’.^100^ In Alexander’s text, there is no attempt to compare this remedy with other similar ones. His usual heuristic approach, as displayed in pharmacology, is absent here. In this case experience is clearly used in an attempt to maintain the effectiveness of his undoubtedly curious therapeutic recommendations.

There is one other remarkable example, in which Alexander abandons any hint of an apology in his words and, by employing his usual strong first-person singular, he makes his presence more obvious than in any other case in his section on natural remedies:

‘I received [ἔλαβον] this in Tuscia from a peasant who claimed to have cured someone of the disease by chance; for he happened to be cutting a wild rue^101^ (….) He came full of the effluvia of the rue, he held his nose, and then he rose up and was no longer seized by the disease. And I have treated [ἐθεράπευσα] (patients) many times (with this remedy). And so you must admire it and consider it excellent and let it not be shared any further’.^102^

First, we are informed about the origin of the remedy, which confirms Alexander’s practical method in getting new material. But before we comment on his method, let us see how Alexander considers he must end his passage. He actually discourages his reader from circulating his remedy. Although the great competition between different kinds of healers at the time must have played an important role in his decision to include natural remedies,^103^ he shows that his initiative in testing the effectiveness of these remedies on his patient proved valuable in a couple of times. This is clearly not an attempt to give a cryptic air to his words, but instead it should be seen as part of Alexander’s attempt to involve his audience more actively by revealing the outcome of his clinical practice. This could function as a further ‘hook’ for the reader, who may still have concerns as regards the nature and efficacy of some remedies.

As regards Alexander’s personal involvement with the remedy, at this point we can see him collecting and testing even material derived from oral sources. This shows his awareness of the need to obtain information from different perspectives, some of which cannot be found in written material. This may be seen as the first step towards the rationalisation of a certain remedy. It is true that a certain natural drug such as the root of a plant may have been used for many centuries in popular healing practices. Thus, although Alexander’s natural remedies are clearly separated from his ‘mainstream’ approach, his anxiety to test them may correspond to a wider concept, in which the accumulated experience of using a certain plant for a long period of time offers a rational explanation leading to the plant perhaps finally being included in the ‘official’ *materia medica*.

## 6. Conclusion

‘I like to use every possible means (in treating my patients)’^104^

This article has brought out two significant aspects in which Alexander’s clinical experience deserves consideration and careful interpretation. In contrast to other late antique medical authors, Alexander shows a remarkable effort to involve in his writing the long contact with the sufferer by presenting critically all that he knows. Alexander is not simply a mediator or a compiler of the most famous remedies and theories of his time. He is an active physician, who having already travelled a great deal and with an awareness of the ancient literature on the subject, tries to evaluate, adjust and adopt earlier therapeutic agents from a broad variety of sources. A strict scientific reasoning might not follow his inquiring attitude, but it shows his concern not just to test but even to furnish a contemporary medical cabinet, notably in the field of pharmacology. Furthermore, clinical testing proves to be the appropriate tool for including the use of popular healing practices, excluded by earlier authors, but presented separately here as natural remedies. Far from being the remnants of old-fashioned Roman medicine, these remedies reflect a lively image of the sixth-century medical milieu. On a textual level, Alexander is the narrator of a medical account, in which his recommendations are presented as the outcome of his rich experience of diseases. As has been demonstrated, Alexander is aware of the novel nature of these parts of his work, which lead him to impose his strong authorial presence and lend legitimacy to his text.

## Footnotes

1. For an introduction to medicine in late antiquity, see Leendert G. Westerink, ‘Philosophy and Medicine in Late Antiquity’, *Janus*, 51 (1964), 169–77; Owsei Temkin, *Hippocrates in a world of Pagans and Christians*(Baltimore, MD: Johns Hopkins University Press, 1991), 213–48; and Vivian Nutton, *Ancient Medicine*, 2nd edn (London: Routledge, 2013), 299–317. It is notable that in the contents of four recent edited volumes devoted to late antiquity, there is not a single chapter dealing with late antique medical literature out of a total of 104 chapters in all. Cf. M. Maas (ed.), *The Cambridge Companion to the Age of Justinian* (Cambridge and New York: Cambridge University Press, 2005); S.F. Johnson (ed.), *Greek Literature in Late Antiquity: Dynamism, Didacticism, Classicism* (Aldershot: Ashgate, 2006); P. Rousseau and J. Raithel (eds), *A Companion to Late Antiquity* (Chichester: Wiley-Blackwell, 2009); and S.F. Johnson (ed.), *Oxford Handbook of Late Antiquity* (Oxford: Oxford University Press, 2012).

2. Vivian Nutton, ‘From Galen to Alexander: Aspects of Medicine and Medical Practice in Late Antiquity’, *Dumbarton Oaks Papers*, 38 (1984), 1–14: 2.

3. The exact dates of late antique authors are rarely known, and the dates given are only approximations following K.-H. Leven (ed.), *Antike Medizin: ein Lexikon*(Munich: Beck, 2005). On Oribasios, see R. de Lucia, ‘Doxographical hints in Oribasius’ *Collectiones medicae*’, in Ph. van der Eijk (ed.), *Ancient Histories of Medicine. Essay in Medical Doxography and Historiography in Classical Antiquity* (Leiden: Brill, 1999), 473–89; on Caelius Aurelianus, see Anna Maria Urso, *Dall’autore al traduttore. Studi sulle Passiones celeres e tardae di Celio Aureliano* (Messina: Edizioni Dr Antonio Sfameni, 1997); and Ph. van der Eijk, ‘Antiquarianism and criticism: Forms and Functions of medical doxography in Methodism (Soranus, Caelius Aurelianus),’ in Ph. J. van der Eijk, *Idem.*, 397–452; on Theodore Priscianus, see Marco Formisano, ‘The “natural” Medicine of Theodorus Priscianus’, *Philologus*, 148 (2004), 126–42; and on the *Medicina Plinii*, see A. Doody, ‘Authorial voice in the *Medicina Plinii*’, in L. Taub and A. Doody (eds), *Authorial Voices in Greco-Roman Technical Writing* (Trier: Wissenschaftlicher Verlag, 2009), 93–105. See also Ph. van der Eijk, ‘Principles and practices of compilation and abbreviation in the medical “encyclopaedias” of late antiquity’, in M. Horster and C. Reitz (eds), *Condensing texts, condensed texts* (Stuttgart: Franz Steiner, 2010), 519–54, who provides a critical, comparative study of Oribasios’, Aetios of Amida’s and Paul of Aegina’s compiling methods. It is important to note that apart from the medical compilations, which constitute the main bulk of late antique medical texts, there are also commentaries by Alexandrian authors such as Stephen (late sixth/early seventh century AD); for an overview of the late antique medical literature with a special emphasis on the Greek production, see Herbert Hunger, *Die hochsprachliche profane Literatur der Byzantiner* (Munich: Beck, 1978), vol. 2, 291–303.

4. The term ‘clinical’ is not connected with a particular ‘healing space’ such as a clinic or a hospital; rather it denotes the daily direct observation of the patient by a physician irrespectively of the healing place.

5. On the rise of Galenism, that is the medical system based on Galen’s theories and practices, in late antiquity, see Oswei Temkin, *Galenism: rise and decline of a medical philosophy*(Ithaca, NY: Cornell University Press, 1973), 51–94.

6. On the reception of Galen in Alexander’s works, see Alessia Guardasole, ‘L’héritage de Galien dans l’oeuvre d’Alexandre de Tralles’, in J. Jouanna and J. Leclant (eds), *Colloque La médecine grecque antique, Cahiers de la Villa ‘Kérylos’*(Paris: Académie des Inscriptions et Belles-Lettres, 2004), 219–34.

7. Agathias, *Histories*, 5.6, Rudolfus Keydell (ed.) (Berlin: De Gruyter, 1967), 171, 3–6. For an overview of Alexander’s biographical details, see Theodor Puschmann, *Alexander von Tralles: Original-Text und**Übersetzung nebst einer einleitenden Abhandlung: ein Beitrag zur Geschichte der Medicin*, (Vienna: Wilhelm Braumüller, 1878–79), vol. I, 75–87; A. Guardasole, ‘Alessandro di Tralle’, in A. Garzya (ed.), *Medici Bizantini*(Torino: Unione tipografico-editrice torinese, 2006), 557–70; and David Langslow, *The Latin Alexander Trallianus* (London: Society for the Promotion of Roman Studies, 2006), 1–4. See also, John Duffy, ‘Byzantine Medicine in the Sixth and Seventh Centuries: Aspects of Teaching and Practice’, *Dumbarton Oaks Papers*, 38 (1984), 21–7: 25–7, who sets out Alexander’s life and work in the light of contemporary society.

8. J.R. Martindale *et al* *The Prosopography of the Later Roman Empire*(Cambridge: Cambridge University Press, 1992), vol. 3B, 1183, Stephanus 1.

9. Martindale, *ibid.*, vol. 3, 88–9, Anthemius 2.

10. Agathias, *Histories,* 5.6, Keydell (ed.), *op. cit.*(note 7), 171–5.

11. The only critical edition of the *Therapeutics* is by Puschmann, *op. cit.*(note 7), vol. 1, 440–617, and vol. 2, 1–585. See Puschmann, *op. cit.* (note 7), vol. 1, 87–108, who provides a discussion of the manuscripts considered in his edition. For a further discussion on the order of the Books, see Barbara Zipser, ‘Die Therapeutica des Alexander Trallianus: ein medizinisches Handbuch und seine Überlieferung’, in R.-M. Piccione and M. Perkams (eds), *Selecta colligere, II. Beiträge zur Technik des Sammelns und Kompilierens griechischer Texte von der Antike bis zum Humanismus* (Alessandria: Dell’Orso, 2005), 211–34, who shows that the last three books of the *Therapeutics* are reversed in some branches of the textual tradition. On the *editio princeps* of the *Therapeutics*, see A. Guardasole, ‘Sur l’editio princeps d’ Alexandre de Tralles’, in V. Boudon-Millot and G. Cobolet (eds), *Lire les médecins grecs à la Renaissance. Aux origines de l’édition médicale. Actes du colloque international de Paris 19–20 septembre 2003* (Paris: Bibliothèques interuniversitaires de médicine, 2005), 323–37.

12. Puschmann, *op. cit.*(note 7), vol. 1, 287–439, and vol. 2, 586–99, respectively.

13. On the Latin reception, see Langslow, *op. cit* (note 7), 37–74. For the Arabic translation, see Puschmann, *op. cit.*(note 7), vol. 1, 92–5; and Manfred Ullmann, *Die Medizin im Islam* (Leiden: Brill, 1970), 85–6. On the Hebrew fragments, see Puschmann, *op. cit.* (note 7), vol. 1, 91.

14. Alexander of Tralles, *Therapeutics*, 1.14, Puschmann (ed.), *op. cit.*(note 7), vol. 1, 535, 4–6.

15. Cf. Puschmann, *op. cit.*(note 7), vol. 1, 107; and Barbara Zipser, ‘Pseudo-Alexander Trallianus, De oculis: Einleitung, Text, Übersetzung und Kommentar’ (unpublished PhD thesis: Universität Heidelberg, 2003).

16. The text is found in one manuscript, Parisinus gr. 2316, dated to the fifteenth century.

17. John Scarborough, ‘The Life and Times of Alexander of Tralles’, *Expedition*, 39, 2 (1997), 51–60: 60.

18. ‘Natural remedies’ refers to Alexander’s ‘φυσικά’, that is a distinct group of Alexander’s therapeutic material comprising popular healing practices such as the use of amulets or incantations. I use the term ‘remedy’ with reference to a particular medical method, either diagnostic or therapeutic. The term ‘recipe’ is used to connote the preparation of a certain composite drug.

19. Alexander of Tralles, *Therapeutics*, 1.15, Puschmann (ed.), *op. cit.*(note 7), vol. 1, 535–75.

20. For a study of epilepsy throughout classical and late antique medical literature, see Oswei Temkin, *The Falling Sickness*(Baltimore, MD: John Hopkins University Press, 1971), 28–81. In particular for late antique and Byzantine medical authors, see Karl-Heinz Leven, ‘Die “unheilige” Krankheit–Epilepsia, Mondsucht und Besessenheit in Byzanz’, *Würzburger medizinhistorische Mitteilungen*, 13 (1995), 17–57; and Georgios Makris, ‘Zur Epilepsie in Byzanz’, *Byzantinische Zeitschrift*, 88 (1995), 363–404.

21. Alexander of Tralles, *On Fevers*, Proem, Puschmann (ed.), *op. cit.*(note 7), vol. 1, 289, 1–12; all translations from Greek are my own. The current rearrangement reflects Puschmann’s edition. There is a reference in the *Therapeutics* to *On Fevers*, confirming that the former was composed later, Alexander of Tralles, *On Fevers*, 2, Puschmann (ed.), *op. cit.* (note 7), vol. 1, 313, 11–12.

22. The word is used extensively by Alexander in various instances in his works; altogether, there are 94 such mentions.

23. On the use of first-person verbs and pronouns in Aetios of Amida’s medical compilation, see A. Debru, ‘La suffocation hystérique chez Galien et Aetius. Réécriture et emprunt du ‘je”, in A. Garzya (ed.), *Tradizione e ecdotica dei testi medici tardoantichi e bizantini*(Naples: D’Auria, 1992), 79–89.

24. Alexander of Tralles, *Therapeutics*, 1.15, Puschmann (ed.), *op. cit.*(note 7), vol. 1, 561, 4.

25. For example, see (θεάομαι) Alexander of Tralles, *Therapeutics*, 1.17, Puschmann (ed.), *op. cit.*(note 7), vol. 1, 605, 15; *idem*, 5.6, vol. 2, 215, 29; (εὑρίσκω) *idem*, 1.14, vol. 1, 533, 9; and *idem*, 2.1, vol. 2, 55, 3; (ὁράω) Alexander of Tralles, *On Fevers*, 5, Puschmann (ed.), *op. cit.* (note 7), vol. 1, 373, 20; and *idem*, 3, vol. 2, 343, 23.

26. A very small weight, 1/24 ounce or 1.137g. Cf. Friedrich Hultsch, *Metrologicorum scriptorium reliquiae*(Leipzig: Teubner, 1864–6), vol. 1, 169–71; and Julius Berendes, *Des Pedanios Dioskurides aus Anazarbos Arzeimittellehre in Fünf Büchern* (Stuttgart: Ferdinard Enke, 1902), 16.

27. Alexander of Tralles, *Therapeutics*, 1.15, Puschmann (ed.), *op. cit.*(note 7), vol. 1, 547, 27.

28. See Geoffrey Lloyd, *The Revolutions of Wisdom*(Berkeley: University of California Press, 1987), 56–78; H. von Staden, ‘Author and authority. Celsus and the construction of a scientific self’, in M. E. Vázquez Buján (ed.), *Tradición e innovación de la medicina latina de la antigüedad y de la Alta Edad Media* (Santiago de Compostela: Servicio de Publicacións e Intercambio Científico da Universidade de Santiago de Compostela, 1994), 103–17; V. Nutton, ‘Galen’s authorial voice: a preliminary enquiry’, in Taub and Doody (eds), *op. cit* (note 3), 53–62; and Laurence Totelin, ‘And to End on a Poetic Note: Galen’s Authorial Strategies in the Pharmacological Books’, *Studies in History and Philosophy of Science Part A*, 43, 2 (2012), 307–15.10.1016/j.shpsa.2011.12.019PMC339642022849032

29. For further examples, see Alexander of Tralles, *Therapeutics*, 1.14, Puschmann (ed.), *op. cit.*(note 7), vol. 1, 523, 6; *idem*, 6, vol. 2, 243, 18; and *idem*, 12, vol. 2, 567, 7–8.

30. See Susan Mattern, *Galen and the Rhetoric of Healing*(Baltimore, MD: John Hopkins University Press, 2008), 138–40, who provides plenty of examples from Galen’s case histories.

31. For the notion of ‘communality’ in connection with Galen, see J. König, ‘Self-promotion and self-effacement in Plutarch’s *Table Talk*’, in F. Klotz and K. Oikonomopoulou (eds), *The Philosopher’s Banquet: Plutarch’s Table Talk in the Intellectual Culture of the Roman Empire*(Oxford: Oxford University Press, 2011), 183–6, who argues for a didactic relationship between author and reader. See also Friedrich Slotty, ‘Die Stellung des Griechischen und anderer idg. Sprachen zu dem soziativen und affektischen Gebrauch des Plurals der ersten Person’, *Indogermanische Forschungen* 45, (1928), 348–63, who applies the term ‘sociative’ to describe the use of the first-person plural among a variety of Greek literary sources.

32. As in the case of other Greek medical authors as well as in Byzantine medical works of later centuries, there is a great variation in manuscripts as regards the arrangement of the title of each paragraph and its actual place in the folio. For example, sometimes paragraphs are not separated in the main text and the titles are given in the margins without denoting the line of the main text to which they correspond. It seems that the titles were often simply provided by scribes or rearranged in accordance with readers’ preferences. This is a topic that can hardly be treated within the limited confines of a footnote.

33. Alexander of Tralles, *Therapeutics*, 1.15, Puschmann (ed.), *op. cit.*(note 7), vol. 1, 551, 11; and *idem*, 1.15, vol. 1, 561, 3.

34. *Ibid*, 1.15, vol. 1, 535–39; *ibid*, 1.15, vol. 1, 539–57; and *ibid*, 1.15, vol. 1, 557–75 respectively.

35. Epilepsy is discussed a good deal by Galen in various parts of his vast corpus. One of the most detailed descriptions of the disease and its various treatments can be found in *On Affected Parts*, 3.8–11, Karl Gottlob Kühn (ed.) (Leipzig: Carl Cnobloch, 1821–33), vol. 8, 168–201; in particular on the various forms of the disease, see *idem*, 3.11, 198–200. See also Rudolph Siegel, *Galen’s System of Physiology and Medicine: An Analysis of his Doctrines and Observations on Blood Flow, Respiration, Tumors and Internal Diseases*(Basel and New York: Karger, 1968), 308–15, who discusses the Galenic physiology of epilepsy.

36. Alexander of Tralles, *Therapeutics*, 1.15, Puschmann (ed.), *op. cit.*(note 7), vol. 1, 537, 14–5; and *idem*, 1.15, vol. 1, 537, 23–4. On Alexander’s humoural pathology, see Giuseppe Ongaro, ‘La patologia epatica nel pensiero di Alessandro di Tralle’, *Rivista di storia della medicina*, 10 (1996), 58–73.

37. For example, see Alexander of Tralles, *Therapeutics*, 1.15, Puschmann (ed.), *op. cit.*(note 7), vol. 1, 543, 18–19; *idem*, 1.15, vol. 1, 547, 6; and *idem*, 1.15, vol. 1, 551, 20. For Galen, see *On Affected Parts*, 3.9, Kühn (ed.), *op. cit* (note 35), vol. 8, 175; and Galen, *On Tremor, Palpitation, Spasm, and Rigor*, 6, Kühn (ed.), *op. cit* (note 35), vol. 7, 608 and 618.

38. Alexander of Tralles, *Therapeutics*, 1.15, Puschmann (ed.), *op. cit.*(note 7), vol. 1, 549, 27–8.

39. This calculation is based on all of the cases of the first-person singular or plural of a verb and of a first-person singular or plural pronoun.

40. On Alexander’s pharmacology, see John Scarborough, ‘Early Byzantine Pharmacology’, *Dumbarton Oaks Papers*, 38 (1984), 213–32: 226–8.

41. Alexander of Tralles, *Therapeutics*, 1.15, Puschmann (ed.), *op. cit.*(note 7), vol. 1, 575, 6–9.

42. *Ibid*, 1.15, vol. 1, 545, 21–3.

43. For example, see Galen, *On the Properties of Foodstuffs*, 3.16, Kühn (ed.), *op. cit* (note 35), vol. 6, 689–96; Georgius Helmreich (ed.) (Leipzig and Berlin: in aedibus B. G. Teubneri, 1923), *CMG* V/4, 2, 349–53.

44. Cf. Galen, *Advice to an Epileptic Boy*, 1–4, Kühn (ed.), *op. cit* (note 35), vol. 11, 357–68.

45. Temkin, *op. cit* (note 20), 78–81.

46. The retrospective identification and subsequent translation of plant names into modern equivalents is not usually regulated by definite botanical and pharmacognostic criteria and, in most cases, is not as straightforward as it seems. On problems of ancient and medieval plant identification, see Jerry Stannard, ‘Hippocratic Pharmacology’, *Bulletin of the History of Medicine*, 35 (1961), 497–518, here 499–502; and J. Riddle, ‘Research procedures in evaluating medieval medicine’, in B. Bowers (ed.), *The Medieval Hospital and Medical Practice*(Aldershot: Ashgate, 2007), 3–17: 8–9. I prefer to give the most usual English translation in the main text, while I provide the Greek term and indicative botanical names in the footnotes; in this case: ‘ὕσσωπος’ (*Satureia graeca* L. /article/back/fn-group/fn/p/mixed-citation/inline-formula *Micromeria graeca* Benth. or *Hyssopus officinalis* L.). For a discussion on the various identifications of hyssop, see Alfred Andrews, ‘Hyssop in the Classical Era’, *Classical Philology*, 56 (1961), 230–48.

47. Alexander of Tralles, *Therapeutics*, 1.15, Puschmann (ed.), *op. cit.*(note 7), vol. 1, 545, 21–3.

48. Aretaeus, *On the Cure of Acute Diseases*, 1.5, Carolus Hude (ed.) (Berlin: in aedibus Academiae Scientiarum, 1958), *CMG* II, 107, 8–10; Soranus’ advice survives through his doxographer Caelius Aurelianus, *On Chronic Diseases*, 1.4, Israel Edward Drabkin (ed.) (Chicago, IL: University of Chicago Press, 1950), 513.

49. For example, Galen acknowledged the importance of purgatives and dedicated further works to dealing specifically with this class of drugs, *On the Power of Purgative Drugs*, Kühn (ed.), *op. cit* (note 35), vol. 11,323–42, in addition to his extensive *On the Composition of Drugs according to Kind*, Kühn (ed.), *op. cit* (note 35), vol. 13, 362–1058. On the place of purgatives in the Galenic corpus, see S. Vogt, ‘Drugs and pharmacology’, in R. J. Hankinson (ed.), *The Cambridge Companion to Galen*(Cambridge: Cambridge University Press), 304–22: 311–2.

50. Alexander of Tralles, *Therapeutics*, 1.15, Puschmann (ed.), *op. cit.*(note 7), vol. 1, 547, 17–18. As regards *theodōrētos*, there are references only by later authors such as Paul of Aegina, see for example *Epitome of Medicine*, 3.46, Johan Ludvig Heiberg (ed.) (Leipzig; Berlin: in aedibus B. G. Teubneri, 1921–24), *CMG* IX/1, 253. According to a thirteenth-century manuscript, Vaticanus Palatinus gr. 199, which contains some chapters of Paul’s work, the passage referring to *theodōrētos* is attributed to the Roman medical author Archigenes (second half of the first century–first half of the second century AD); cf. Cesare Brescia, *Frammenti medicinali di Archigene* (Napoli: Libreria scientifica editrice, 1950), 20–4; and Aimilios Mavroudis, ᾿Αρχιγένης Φιλίππου ᾿Απαμεύς. ῾Ο βίος καὶ τὰ ἔργα ἑνὸς ῞Ελληνα γιατροῦ στὴν αὐτοκρατορικὴ ῾Ρώμη (Athens: Akadēmia Athēnōn, 2000), 222–3. Alexander himself uses the antidote in two more instances, Alexander of Tralles, *Therapeutics*, 8.2, Puschmann (ed.), *op. cit.* (note 7), vol. 2, 371; and *idem*, 9.3, vol. 2, 419.

51. One ounce is equal to 27.288 g.; cf. Friedrich Hultsch, *op. cit* (note 26), vol. 1, 248–9; and Berendes, *op. cit* (note 26), 16.

52. ‘ἀλόη’ (*Aloe vera* L.).

53. ‘σκαμμωνία’; the resinous exudation from the living root of *Convolvulus scammonia* L.

54. ‘κόμμι’; it probably refers to gum arabic obtained from *Acacia arabica* Wild.

55. ‘κολοκυνθίς’ (*Citrullus colocynthis* Schrad.).

56. ‘βδέλλιον’; the aromatic gum obtained from *Commiphora mukul* Engl.

57. Alexander of Tralles, *Therapeutics*, 1.15, Puschmann (ed.), *op. cit.*(note 7), vol. 1, 547, 18–25. On recipe format in the ancient world, see Cajus Fabricius, *Galens Exzerpte aus älteren Pharmakologen* (Berlin: De Gruyter, 1972), 24–30; and Laurence Totelin, *Hippocratic Recipes: Oral and Written Transmission of Pharmacological Knowledge in Fifth- and Fourth-Century Greece* (Leiden: Brill, 2009), 47–61.

58. On the notion of *diorismos* according to the Empiricists, see Michael Frede, ‘The Empiricist Attitude to Reason and Theory’, *Apeiron*, 21 (1988), 79–97. On Galen, see Ph. van der Eijk, ‘Galen’s use of the concept of “qualified experience” in his dietetic and pharmacological works’, in A. Debru (ed.), *Galen on pharmacology*(Leiden: Brill, 1997), 35–57.

59. ‘λεπίδιον’ (*Lepidium latifolium* L.).

60. Alexander of Tralles, *Therapeutics*, 1.15, Puschmann (ed.), *op. cit.*(note 7), vol. 1, 551, 17–26.

61. Cf. Galen, *On Affected Parts*, 3.11, Kühn (ed.), *op. cit* (note 35), vol. 8, 194 and 198. On Galenic case histories see, Luis García Ballester, ‘Elementos para la construcción de las historias clínicas en Galeno’, *Dynamis*, 15 (1995), 47–65; and Cristina Álvarez-Millán, ‘Greco-Roman Case Histories and Their Influence on Islamic Clinical Accounts’, *Social History of Medicine*, 12 (1999), 19–43: 30–3. See also, Mattern, *op. cit* (note 30), who provides a fresh study of the entire group of Galenic case histories. In particular, on Galen’s young patients, see Danielle Gourevitch, *I giovani pazienti di Galeno: per una patocenosi dell’impero romano*(Roma: Laterza, 2001).

62. ‘θαψία’ (*Thapsia garganica* L.).

63. ‘νᾶπυ’ (*Sinapis alba* L.).

64. See Mattern, *op. cit* (note 30), 40–3, where examples of borrowing of case histories are presented.

65. Galen, *On the Powers of Simple Drugs*, 8.8, Kühn (ed.), *op. cit* (note 35), vol. 12, 58, 1–4. Later on, the same action is mentioned by Oribasios, *Medical Collections*, 15.1.11, Johann Raeder (ed.) (Leipzig; Berlin: in aedibus B.G. Teubneri, 1929), *CMG* VI/1, 2, 262, 7–8; and Aetios of Amida, *Sixteen Books*, 1.249, Alexander Olivieri (ed.) (Leipzig; Berlin: in aedibus B.G. Teubneri, 1935), *CMG*VIII/1, 102, 25, who both quote Galen’s passage.

66. For the notion of experiment in an ancient environment and, in particular, in Hellenistic medicine, see Heinrich von Staden, ‘Experiment and Experience in Hellenistic Medicine’, *Bulletin of the Institute of Classical Studies*, 22 (1975), 178–99.21213700

67. ‘ἑλλέβορος λευκός’ (*Veratrum album* L.).

68. On Methodism, see M. Frede, ‘The Method of the So-Called Methodical School of Medicine’, in M. Frede (ed.), *Essays in Ancient Philosophy*(Minneapolis, MN: University of Minnesota Press, 1987), 261–78. In particular on the therapy of epilepsy, see Temkin, *op. cit* (note 20), 74–6.

69. Caelius Aurelianus, *On Chronic Diseases*, 1.4, Drabkin (ed.), *op. cit* (note 48), 503ff.

70. A popular purgative, usually aloe based. Alexander in his chapter ‘On Paresis’, Alexander of Tralles, *Therapeutics*, 1.16, Puschmann (ed.), *op. cit.*(note 7), vol. 1, 581, gives a long recipe for a *hiera* made up of 24 ingredients; cf. Galen, *On the Composition of Drugs according to Places*, 9.2, Kühn, *op. cit* (note 35), vol. 13, 136–7.

71. See the following passage from Alexander of Tralles, *On Fevers*, 7, Puschmann (ed.), *op. cit.*(note 7), vol. 1, 429, 16–431, 14, where Alexander gives detailed instructions for the preparation of the Armenian stone, a drug of mineral origin; cf. Dioscorides, *De materia medica*, 5.90, Max Wellmann (ed.) (Berlin: Weidmann, 1914), vol. 3, 63, 16–21; Dietlinde Goltz, *Studien zur Geschichte der Mineralnamen in Pharmazie, Chemie und Medizin von den Anf**den Anfängen bis Paracelsus* (Wiesbaden: F. Steiner, 1972), 146–7; and Scarborough, *op. cit* (note 40), 227–8, who argues that it is a combination of copper oxyacetate, azurite (/article/back/fn-group/fn/p/mixed-citation/inline-formula  and malachite /article/back/fn-group/fn/p/mixed-citation/inline-formula

72. Alexander of Tralles, *Therapeutics*, 1.15, Puschmann (ed.), *op. cit.*(note 7), vol. 1, 555, 24–557, 3.

73. That is ‘ἡ τοῦ ὁμοίου μετάβασις’; the other two main branches were ‘ἐμπειρία’ (experience) and ‘ἱστορία’ (history); for a discussion, see von Staden, *op. cit* (note 66), 191–2.

74. The etymology and the use of the term ‘φυσικά’ by Alexander to describe part of his remedies is a complicated issue and not within the scope of this article. I have chosen to translate the word ‘φυσικά’ as ‘natural remedies’, thus keeping the literal meaning of the word, rather than using ‘magical’ or ‘occult’, which might have suggested the notion of a divine or mystic intervention to the modern reader. See Henry G. Liddell, Robert Scott and Henry S. Jones (eds), *A Greek–English Lexicon*, 9th edn (Oxford: Clarendon Press, 1996), s.v. φυσικός: belonging to occult laws of nature, magical, φάρμακα spells or amulets. Note also that this particular term has been translated into German as ‘Wundermittel’, Alexander of Tralles, *Therapeutics*, 1.15, Puschmann (ed.), *op. cit.*(note 7), vol. 1, 556, 14; into French as ‘naturels’, A. Guardasole, ‘Alexandre de Tralles et les remèdes naturels’, in F. Collard and E. Samama (eds), *Mires, physiciens, barbiers et charlatans: les marges de la médicine de l’Antiquité au XVIe siècle* (Langres: D. Gueniot, 2004), 81–99: 81; and into Italian as ‘magici’, Guardasole, *op. cit.* (note 7), 663. Moreover, the following terms have been used in English: ‘natural’ and ‘occult’ by Temkin, *op. cit* (note 20), 25 and *op. cit.* (note 1), 233, respectively; ‘magical’ by John Duffy ‘Reactions of two Byzantine intellectuals to the theory and practice of magic: Michael Psellos and Michael Italikos’, in H. Maguire (ed.), *Byzantine Magic* (Washington, DC: Dumbarton Oaks Research Library and Collection, 1995), 83–97: 95; Scarborough, *op. cit* (note 17), 55; and P. Horden ‘Sickness and healing’, in T. Noble, J. Smith and R. Baranowski (eds), *Early Medieval Christianities. c.600–c.1100* (Cambridge: Cambridge University Press, 2008), 416–32: 425; and ‘sympathetic’ by Ludwig Eldestein, ‘Greek Medicine in its Relation to Religion and Magic’, *Bulletin of the Institute of the History of medicine*, 5 (1937), 201–46: 235; and Nutton, *op. cit* (note 2), 8.

75. Although Galen is not always as rational as some have believed, his support for the use of amulets or incantations, in the sense in which Alexander refers to them, seems unlikely. On Galen’s criticism, see Galen, *On the Powers of Simple Drugs*, 7.proem and 7.10, Kühn (ed.), *op. cit* (note 35), vol. 11, 792–3, and 859. See also John Scarborough, *Roman Medicine*(London: Thames and Hudson, 1969), 119–20, who provides examples of Galen’s partial acceptance of remedies with superstitious connotations; Vivian Nutton, ‘From Medical Certainty to Medical Amulets: Three Aspects of Ancient Therapeutics’, *Clio medica*, 22 (1991), 13–22; H. von Staden, ‘Galen’s daimon. Reflections on ‘rational’ and ‘irrational’, in N. Palmieri (ed.) *Rationnel et irrationnel dans la mâedecine ancienne et mâediâevale: aspects historiques, scientifiques et culturels* (Saint-Etienne: Publications de l’universitâe de Saint-Etienne, 2003), 15–44; and Jacques Jouanna, ‘Médecine rationnelle et magie: le statut des amulettes et des incantations chez Galien’, *Revue des études grecques*, 124 (2011), 47–77. Soranus refers to lots of people using amulets, which do not produce any significant effect, but they might help the patients; cf. Soranus, *Gynaecology*, 3.42, Ioannes Ilberg (ed.) (Leipzig and Berlin: in aedibus B. G. Teubneri, 1927), *CMG* IV, 121, 26–31. For an overview of the views of Latin medical authors, see William H. S. Jones, ‘Ancient Roman Folk Medicine’, *Journal of the History of Medicine and Allied Sciences*, 12 (1957), 459–72; and Ugo Capitani, ‘Celso, Scribonio Largo, Plinio il Vecchio e il loro atteggiamento nei confronti della medicina popolare’, *Maia*, 24 (1972), 120–40.11636800

76. On Archigenes, see Alexander’s references as regards epilepsy: Alexander of Tralles, *Therapeutics*, 1.15, Puschmann (ed.), *op. cit.*(note 7), vol. 1, 557, 18–23; *idem*, 1.15, 561, 19; *idem*, 1.15, 563, 4; and *idem*, 1.15, 567, 10; see also Mavroudis, *op. cit* (note 50), 50.

77. It is important to note that although a great number of natural remedies are included in Aetios of Amida’s late antique medical compilation as well, these are listed along with the discussion of ‘mainstream’ remedies and the author does not intend to separate them. For a discussion of Aetios of Amida’s references to popular medicine, see Giovanni Mercatti, ‘Un nuovo capitolo di Sorano e tre nuove ricette superstiziose in Aezio’, *Studi e Testi*, 31 (1917), 42–6; and Irene Calà, ‘Per l’edizione del primo dei *Libri medicinales* di Aezio Amideno’ (unpublished PhD thesis: Università di Bologna, 2012), 48–52.

78. On Marcellus’ pharmacology, see Jerry Stannard, ‘Marcellus of Bordeaux and the Beginning of Medieval Materia Medica’, *Pharmacy in History*, 15 (1973), 47–53.11609463

79. We know that special works on *physika* have been written by atuhors such as Bolos (ca. 200 BC) and Aelius Promotus (first/second century AD), which are now lost; cf. Theodor Weidlich, *Die Sympathie in der antiken Litteratur*(Stuttgart: K. Hofbuchdruckerei C. Liebich, 1894), 11–75; and Max Wellmann, ‘Die Φυσικά des Bolos Demokritos und der Magier Anaxilaos aus Larissa’, *Abhandlungen der Preussischen Akademie der Wissenschaften: Philosophisch-historische Klasse*, 7 (1928), 1–80.

80. In the *Therapeutics* there are, in addition to the chapter on epilepsy, four more cases of natural remedies being described in the following chapters: (i) ‘On Hiccups’, Alexander of Tralles, *Therapeutics*, 7.9, Puschmann (ed.), *op. cit.*(note 7), vol. 2, 319, 12–28); (ii) ‘On Colic’, *idem*, 8.2, vol. 2, 375, 20–377, 31; (iii) ‘On Nephritic Diseases’, *idem*, 11.1, vol. 2, 475, 18–24; and (iv) ‘On Gout’, *idem*, 12, vol. 2, 579, 13–585, 24. Furthermore, we have two more cases in *On Fevers*: (i) ‘On Quotidian Fever’, *idem*, 6, vol. 1, 407, 1–10; and (ii) ‘On Quartan Fever’, *idem*, 7, vol. 1, 437, 5–24. For a recent overview of Alexander’s natural remedies, see Guardasole, *Remèdes naturels*, *op. cit* (note 74), 81–99. See also A. Garzya, ‘Science et conscience dans la pratique médicale de l’Antiquité tardive et byzantine’, in H. Flashar and J. Jouanna (eds), *Médecine et morale dans l’Antiquité: dix exposés suivis de discussions, Vandoeuvres-Genève, 10–23 août 1996* (Geneva: Fondation Hardt, 1997), 337–63, who discusses the concept of conscience in late antique medicine.

81. Alexander of Tralles, *Therapeutics*, 1.15, Puschmann (ed.), *op. cit.*(note 7), vol. 1, 567, 11. On Asklepiades Pharmakion, see A. Touwaide, ‘Asklēpiadēs Pharmakiōn’, in P. T. Keyser and G. L. Irby-Massie (eds), *Encyclopedia of Ancient Natural Scientists* (London: Routledge, 2008), 169–70; Leven (ed.), *op. cit* (note 3), 550.

82. Alexander of Tralles, *Therapeutics*, 1.15, Puschmann (ed.), *op. cit.*(note 7), vol. 1, 569, 2. On Damokrates, see S. Vogt, ‘Seruilius Dāmokratēs’ in Keyser and Irby-Massie (eds), *ibid*, 226.

83. Alexander of Tralles, *Therapeutics*, 1.15, Puschmann (ed.), *op. cit.*(note 7), vol. 1, 561, 2. On Xenokrates, see A. Zucker, ‘Xenokrats of Aphrodisias (Kilikia),’ in Keyser and Irby-Massie (eds), *op. cit* (note 81), 836–7.

84. Alexander of Tralles, *Therapeutics*, 1.15, Puschmann (ed.), *op. cit.*(note 7), vol. 1, 559, 17. On Theodore Priscianus’ references to *physika*, see A. Fraisse, ‘Médecine rationnelle et irrationnelle dans le livre I des Euporista de Théodore Priscien’, in Palmieri (ed.), *op. cit* (note 75), 183–92.

85. Alexander of Tralles, *Therapeutics*, 1.15, Puschmann (ed.), *op. cit.*(note 7), vol. 1, 563, 6; and *idem*, 1.15, vol. 1, 565, 11. On Straton, see A. Touwaide, ‘Strat/article/back/fn-group/fn/p/mixed-citation/inline-formula n of B/article/back/fn-group/fn/p/mixed-citation/inline-formula rutos’, in Keyser and Irby-Massie (eds), *op. cit* (note 81), 765.

86. Alexander of Tralles, *Therapeutics*, 1.15, Puschmann (ed.), *op. cit.*(note 7), vol. 1, 563, 17. On Moschion, see J. Scarborough, ‘Moskhin,’ in Keyser and Irby-Massie (eds), *op. cit* (note 81), 563.

87. Alexander of Tralles, *Therapeutics*, 1.15, Puschmann (ed.), *op. cit.*(note 7), vol. 1, 565, 7. On Marsinos, see P.T. Keyser, ‘Marsinus of Thrak/article/back/fn-group/fn/p/mixed-citation/inline-formula ’, in Keyser and Irby-Massie (eds), *op. cit* (note 81), 534.

88. Alexander of Tralles, *Therapeutics*, 1.15, Puschmann (ed.), *op. cit.*(note 7), vol. 1, 567, 16. On Osthanes, see A. Panaino, ‘Ostan/article/back/fn-group/fn/p/mixed-citation/inline-formula s, pseudo’, in Keyser and Irby-Massie (eds), *op. cit* (note 81), 599–600.

89. Alexander of Tralles, *Therapeutics*, 1.15, Puschmann (ed.), *op. cit.*(note 7), vol. 1, 567, 13. On Zalachthes, see A.Y. Reed, ‘Zakhalias of Babyl/article/back/fn-group/fn/p/mixed-citation/inline-formula n’, in Keyser and Irby-Massie (eds), *op. cit* (note 81), 843.

90. Alexander of Tralles, *Therapeutics*, 1.15, Puschmann (ed.), *op. cit.*(note 7), vol. 1, 563, 11.

91. *Ibid*, 1.15, vol. 1, 565, 1.

92. *Ibid*, 1.15, vol. 1, 565, 4.

93. *Ibid*, 1.15, vol. 1, 565, 16.

94. Alexander of Tralles, *Therapeutics*, 1.15, Puschmann (ed.), *op. cit.* (note 7), vol. 1, 557, 12–8.

95. *Ibid*, 7.9, vol. 2, 319, 4–8.

96. See Temkin, *op. cit* (note 20), 15–21, who discusses epilepsy and demonic possession.

97. Alexander of Tralles, *Therapeutics*, 1.15, Puschmann (ed.), *op. cit.*(note 7), vol. 1, 567, 9; and *idem*, 1.15, vol. 1, 567, 21. These short statements on the efficacy of a particular remedy may be found in medical texts from antiquity to the Middle Ages. See Clair Jones, ‘Efficacy Phrases in Medieval English Medical manuscripts’, *Neuphilologisches Mitteilungen*, 99 (1998), 199–209, who calls them ‘efficacy phrases’; and Laurence Totelin, ‘Old Recipes, New Practice? The Latin Adaptations of the Hippocratic Gynaecological Treatises’, *Social History of Medicine*, 24, 1 (2011), 74–91, who discusses the notion of efficacy in Latin texts.

98. Aretaeus, *On the Cure of Chronic Diseases*, 1.4, Hude (ed.), *op. cit* (note 48), *CMG* II, 154, 6–8. Celsus, *On Medicine*, 3.23, Friedrich Marx (ed.) (Leipzig; Berlin: in aedibus B. G. Teubneri, 1915), *CML*I, 139–40.

99. Scribonius Largus, *Compositiones*, 17, Sergio Sconocchia (ed.) (Leipzig; B. G. Teubner, 1983), 20, 26–30.

100. Alexander of Tralles, *Therapeutics*, 1.15, Puschmann (ed.), *op. cit.*(note 7), vol. 1, 565, 10.

101. “πήγανον ἄγριον” (*Peganum harmala* L.).

102. Alexander of Tralles, *Therapeutics*, 1.15, Puschmann (ed.), *op. cit.*(note 7), vol. 1, 563, 11–16.

103. For example, see *ibid*, 8.2, vol. 2, 375, 10–16, where he refers to a group of wealthy patients who seem to refuse a painful method of purgation that uses enemas and prefer to cure their pains with the help of amulets.

104. Alexander of Tralles, *Therapeutics*, 1.15, Puschmann (ed.), *op. cit.*(note 7), vol. 1, 573, 1.

105. I use the term ‘paresis’ for Alexander’s ‘πάρεσις’, which denotes loss of the ability to move a particular part of the human body in contrast to the term ‘ἀποπληξία’, which is mentioned as a total impairment of the ability to move any part of the human body; cf. Alexander of Tralles, *Therapeutics*, 1.15, Puschmann (ed.), *op. cit.*(note 7), vol. 1, 575, 11–19. For a discussion of the various terms used in ancient medical literature, which define various paralytic conditions, see A. Karenberg, ‘Lähmung’, in Leven (ed.), *op. cit* (note 3), 550.

